# Precision in Practice: Clinical Indication-Specific DRLs for Head CT for Advanced Personalised Dose Benchmarking

**DOI:** 10.3390/diagnostics15151849

**Published:** 2025-07-23

**Authors:** Nora Almuqbil, Zuhal Y. Hamd, Wiam Elshami, Mohamed Abuzaid

**Affiliations:** 1 Department of Radiological Sciences, College of Health and Rehabilitation Sciences, Princess Nourah bint Abdulrahman University, P.O. Box 84428, Riyadh 11671, Saudi Arabia; noaalmuqbil@pnu.edu.sa (N.A.); zyhamd@pnu.edu.sa (Z.Y.H.); 2 Medical Diagnostic Imaging Department, College of Health Sciences, University of Sharjah, Sharjah P.O. Box 27272, United Arab Emirates; welshami@sharjah.ac.ae; 3 Research Institute for Medical and Health Sciences, University of Sharjah, Sharjah P.O. Box 27272, United Arab Emirates

**Keywords:** clinical indication, diagnostic reference levels, computed tomography, radiation dose optimisation, CTDIvol, DLP

## Abstract

**Background/Objectives:** Computed tomography (CT) of the head is vital in diagnosing neurological conditions but poses concerns regarding radiation exposure. Traditional diagnostic reference levels (DRLs) are based on anatomical regions, potentially overlooking variations in radiation requirements driven by clinical indication. This study aimed to establish clinical indication-specific DRLs (DRLCIs) for adult head CT to support precision benchmarking and optimise patient safety. **Methods**: A retrospective observational study was conducted using data from 378 adult patients undergoing non-contrast CT head scans between September 2022 and February 2024. Data on patient demographics, protocols, and radiation dose metrics (Computed Tomography Dose Index Volume and Dose–Length Product) were extracted using DoseWatch™ software. Protocol parameters were standardised across clinical indications such as trauma, stroke, headache, seizure, and infection. Descriptive statistics and correlation analyses were performed. Descriptive statistics, including means, standard deviations, and percentile distributions, were calculated. Correlation analyses were conducted using Pearson’s correlation coefficient to examine relationships between dose metrics and patient variables such as age and body mass index. **Results**: Mean CTDIvol values ranged from 50.58 mGy (trauma) to 52.90 mGy (infection), while DLP values ranged from 1052.52 to 1219.98 mGy·cm. Percentile distributions were narrow, indicating effective protocol standardisation. The strongest correlation was observed between CTDIvol and DLP (r = 0.89), while age and body mass index showed negligible influence on dose metrics. Comparative analysis showed alignment with international benchmarks from the UK, Qatar, Bahrain, and Nigeria. **Conclusions**: This study establishes DRLCIs for adult head CT, demonstrating consistent radiation dose delivery across indications with minimal variability. Clinical indication-based benchmarking enhances dose optimisation and aligns with global radiological protection frameworks.

## 1. Introduction

Computed tomography (CT) of the head is a cornerstone of diagnostic imaging, widely utilised for evaluating a spectrum of clinical indications, including acute stroke, traumatic brain injury, suspected intracranial haemorrhage, tumours, and vascular abnormalities [1,2]. Its diagnostic power, however, comes with the responsibility to manage radiation exposure, as CT contributes significantly to medical radiation doses received by patients. The principle of optimization in radiation protection, as outlined by the International Commission on Radiological Protection (ICRP), emphasises delivering the minimum radiation dose necessary to achieve diagnostic-quality images [3]. Diagnostic Reference Levels (DRLs) are critical tools in this endeavour, serving as benchmarks to guide facilities in assessing and optimising radiation doses without compromising image quality. Two standard dose metrics commonly used in CT imaging are the Computed Tomography Dose Index Volume (CTDIvol) and Dose–Length Product (DLP). CTDIvol represents the average radiation dose delivered per slice in a standardised phantom, while DLP reflects the total radiation dose over the length of the scan. These metrics enable comparison of radiation doses across clinical indications and institutions. Traditionally, DRLs for head CT have been established based on anatomical regions, typically reported as the 75th percentile of dose distributions (e.g., CTDIvol and DLP) for standard protocols [4,5]. However, this approach often overlooks the variability in radiation dose requirements driven by differing clinical indications, which may necessitate distinct imaging protocols [6,7,8].

The rationale for developing clinical indication specific DRLs (DRLCIs) stems from the recognition that radiation dose requirements vary significantly depending on the diagnostic purpose of the CT examination. For instance, a non-contrast CT for acute stroke demands different technical parameters compared to a contrast-enhanced CT for tumour evaluation or a CT angiography (CTA) for aneurysm detection [9,10,11,12]. These variations influence dose metrics such as the Computed Tomography Dose Index (CTDIvol) and Dose–Length Product (DLP), which are standard measures of radiation output and total dose, respectively. Generic DRLs, while useful, may not adequately reflect the nuanced dose requirements of specific clinical scenarios, potentially leading to over- or under-exposure. Over-exposure increases patient risk without diagnostic benefit, while under-exposure may compromise image quality, necessitating repeat scans and further radiation exposure. By tailoring DRLs to clinical indications, radiology departments can achieve more precise dose benchmarking, aligning with the ICRP’s “as low as reasonably achievable” (ALARA) principle and enhancing patient safety [13,14,15].

Multiple international efforts have validated this approach. The European Commission’s EUCLID project, for example, highlighted the shortcomings of anatomical DRLs and proposed the development of DRLs based on common clinical indications across Europe [16]. Similarly, studies from Bahrain and Qatar revealed dose variations within the same anatomical region based on clinical context, supporting the need for clinical indication-based stratification [11,17].

Despite the increasing emphasis on dose optimization, there is a paucity of comprehensive data on clinical indication-specific DRLs for head CT. Existing DRLs, such as those reported by European national surveys, are often generalised across all head CT examinations, with limited stratification by clinical indication [18]. This gap hinders the ability of radiology facilities to benchmark their doses against peers for specific clinical scenarios, potentially leading to inconsistent practices and suboptimal patient outcomes.

This study addresses these challenges by determining and establishing clinical indication-based DRLs for adult head CT examinations. By analysing radiation dose metrics (CTDIvol and DLP) across various clinical scenarios, we seek to provide a framework for advanced personalised dose benchmarking [19]. This study aimed to establish clinical indication-based DRLs for CT head examinations. It adds evidence supporting the need for tailored dose benchmarks to enhance optimisation and reflect real-world clinical practice.

## 2. Materials and Methods

### 2.1. Study Design and Data Collection

Data for this study were collected retrospectively to evaluate radiation dose metrics in adult patients undergoing CT head examinations. This observational retrospective cohort study evaluated radiation doses associated with CT head examinations conducted between September 2022 and February 2024. Patients were identified through the Picture Archiving and Communication System (PACS), which enabled the selection of relevant CT head studies. Clinical data including patient demographics, examination protocols, and radiation exposure parameters were extracted for data analysis.

A total of 378 adult patient records were selected based on the availability of complete dose measurement data obtained through General Electric’s DoseWatch™ software, (General Electric Healthcare, Waukesha, WI, USA). The inclusion criteria for this study were adult patients (aged 18 years and above) who underwent non-contrast CT head examinations between September 2022 and February 2024, with complete data available on radiation dose metrics (CTDIvol and DLP) and patient demographics (age, gender, weight, and height). Exclusion criteria included paediatric patients (under 18 years of age), examinations involving intravenous contrast media, and cases with missing or incomplete dosimetric or demographic data. Given this study’s retrospective nature, the institutional review board waived the requirement for informed consent.

### 2.2. CT Equipment

All data were acquired using an Optima CT660 CT scanner (GE Healthcare, Milwaukee, WI, USA). The data were collected using DoseWatch software (GE Healthcare) for CT dose management. The CT scanner was subjected to routine daily calibration and periodic quality assurance checks in accordance with institutional standards, ensuring consistent performance and accurate dosimetric measurements throughout the study period.

### 2.3. Protocol Parameters Based on Clinical Indications

This study included protocol specifications for routine brain imaging. The clinical indications comprised mental status changes, trauma, stroke, falls, and suspected intracranial haemorrhage. Proper head positioning was ensured by aligning a line from the lateral canthus of the eye to the external auditory canal (EAC) perpendicular to the CT tabletop. In all cases, metallic and high-density objects were removed from the scan area.

Table 1 outlines the technical parameters for the CT head scan protocol used in this study. The scan is performed using a helical technique with 32 detector rows with the scan field of view (FOV) set specifically to the head, with a pitch value of 0.531, indicating closely spaced helical acquisition for high-resolution imaging. The tube voltage is fixed at 120 kV. Automatic Exposure Control (AEC) is employed using the “Smart mA” system, allowing tube current modulation between 130 and 440 mA, while the manually set milliampere is 210 mA. Images are reconstructed with a 5 mm slice thickness and 5 mm interval.

### 2.4. Data Analysis

Data were analysed using IBM SPSS Version 26 (New Orchard Road, Armonk, New York, NY, USA). Descriptive statistics were used to summarise gender, BMI categories, and clinical indications. Continuous variables such as age, BMI, CTDIvol, and DLP were reported as means, standard deviations, and percentiles (25th, 50th, and 75th). Radiation dose indices were compared across clinical indications to identify variation in exposure. Correlation analysis using Pearson coefficients was conducted to assess relationships between age, BMI, CTDIvol, and DLP.

## 3. Results

### 3.1. Demographic and Clinical Indication Profile of CT Head Patients

Table 2 provides a summary of the demographic distribution and clinical indications for patients undergoing CT head examinations. Among the 378 individuals included, males represented a slightly higher proportion at 55.6% (*n* = 210), compared to females at 44.4% (*n* = 168). In terms of body mass index (BMI), most patients were classified as overweight (47.4%, *n* = 179), followed by those with a healthy weight range of 18.5–24.9 kg/m^2^ (41.0%, *n* = 155), while a smaller proportion were obese (11.6%, *n* = 44). Regarding clinical indications for CT imaging, trauma accounted for the most frequent reason (27.0%, *n* = 102), closely followed by headache and stroke, each comprising 26.2% of the cases (*n* = 99, respectively). Seizures were the indication in 11.4% of the patients (*n* = 43), and infection was noted in 9.3% (*n* = 35). This distribution highlights the predominance of trauma-related and neurological presentations in CT head referrals, with a notable overlap between patient weight categories and imaging indications.

### 3.2. Radiation Dose Parameters by Clinical Indication for CT Head Examinations

Table 3 presents the mean, standard deviation (SD), and key percentile values for radiation dose indices—CTDIvol (mGy) and DLP (mGy·cm)—across five clinical indications for CT head examinations. Among all indications, infection-related scans demonstrated the highest mean CTDIvol (52.90 mGy) and DLP (1142.97 mGy·cm), suggesting a marginally elevated radiation burden, possibly due to more complex diagnostic requirements. In contrast, trauma cases exhibited the lowest mean CTDIvol (50.58 mGy) and DLP (1052.52 mGy·cm), indicating more optimised dose use in routine emergency protocols. The headache, seizure, and stroke categories showed relatively consistent dose distributions, with CTDIvol means ranging from 50.65 to 51.88 mGy and corresponding DLP values generally between 1065.02 and 1099.97 mGy·cm. Notably, stroke examinations had a slightly lower mean CTDIvol than seizure and headache but still fell within a narrow interquartile range. The consistency in 25th to 75th percentile values across all indications reflects effective standardisation of protocols and indicates that dose delivery remains tightly controlled. A one-way ANOVA was performed to assess whether differences in CTDIvol and DLP across the clinical indications were statistically significant. The results showed a statistically significant difference in both CTDIvol (*p* = 0.043) and DLP (*p* = 0.038) between the clinical groups. Post hoc analysis (Tukey HSD) revealed that infection-related scans had significantly higher dose values compared to trauma and stroke indications (*p* < 0.05), while other comparisons were not statistically significant. These results support the feasibility of establishing clinical indication specific DRLs and further highlight the value of protocol adherence in minimising dose variability while maintaining diagnostic quality.

### 3.3. Correlation Analysis Between Patient and Dose Parameters

**Figure 1:**
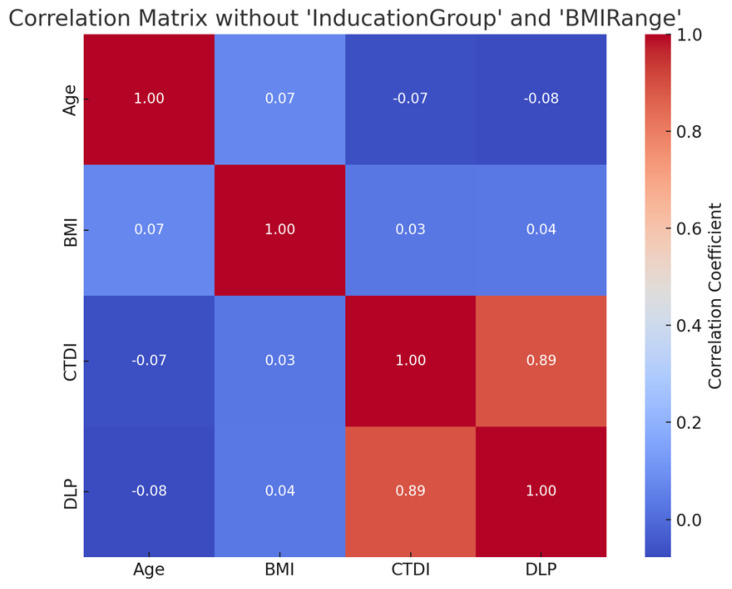
Correlation matrix of continuous variables showing Pearson correlation coefficients between age, BMI, CTDIvol, and DLP. Strong positive correlation is observed between CTDIvol and DLP (r = 0.89), while other relationships are negligible.

Figure 1 displays the correlation matrix examining relationships among age, BMI, CTDIvol, and DLP in CT head examinations, excluding the categorical variables ‘IndicationGroup’ and ‘BMIRange’. The strongest observed correlation is between CTDIvol and DLP (r = 0.89), reflecting a direct and expected association between these two radiation dose metrics. Conversely, correlations between demographic factors (age and BMI) and dose indices are weak and near zero. Age showed a slight negative correlation with both CTDIvol (r = −0.07) and DLP (r = −0.08), while BMI had marginal positive correlations with CTDIvol (r = 0.03) and DLP (r = 0.04). These findings suggest that within this dataset, variations in patient age and BMI had minimal influence on the delivered radiation dose, and protocol standardisation likely contributed to consistent exposure across different patient profiles.

## 4. Discussion

This study provides a focused analysis of radiation dose metrics in adult head CT examinations by establishing clinical indication-specific Diagnostic Reference Levels (DRLCIs), a step forward in advancing patient-centred dose optimisation. Traditionally, DRLs have been based on anatomical regions, providing general benchmarks that may overlook nuanced variations in clinical need. Our findings underscore the importance of stratifying DRLs by clinical indication to better align radiation dose with diagnostic requirements, as advocated by international bodies such as the International Commission on Radiological Protection (ICRP) and the European Commission’s EUCLID project [3,16].

The analysis of 378 adult patients revealed that trauma, headache, and stroke were the most frequent clinical indications for head CT, collectively accounting for nearly 80% of cases. Despite this diagnostic diversity, the distribution of dose parameters—CTDIvol and DLP—remained relatively consistent across these indications, suggesting strong adherence to standardised protocols. Infection-related cases exhibited slightly higher mean doses, potentially due to the need for finer resolution or longer scan coverage in complex pathology. These findings emphasise the necessity of indication-specific benchmarks that reflect real-world diagnostic demands rather than relying on one-size-fits-all anatomical DRLs.

Importantly, the observed 25th to 75th percentile ranges for CTDIvol and DLP were narrow across all clinical indications. This indicates low variability in radiation practices and highlights the maturity of protocol optimisation within the institution. Moreover, the correlation matrix showed a strong positive relationship between CTDIvol and DLP (r = 0.89), as expected, since these are directly linked dose metrics. Conversely, the weak or negligible correlations between patient-specific variables (age and BMI) and radiation dose metrics suggest that protocol-driven dose control, particularly with automatic exposure modulation, is effectively standardising output across diverse patient demographics.

This study’s methodology, which excluded paediatric and contrast-enhanced examinations, ensured homogeneity in the protocol and patient population. This allowed for a more accurate characterisation of DRLCIs, though it also limits generalisability to broader clinical settings. Contrast-enhanced scans, which are routine in vascular and oncologic imaging, should be considered in future work to establish comprehensive DRLCI datasets. Additionally, while our data were obtained using a single CT scanner model (GE Optima CT660), this provides a controlled setting for analysis but may restrict extrapolation across other scanner technologies or institutions.

Another key observation is that the average CTDIvol and DLP values in this study are consistent with international DRL benchmarks reported by the UK’s National Diagnostic Reference Levels (NDRLs) and studies in Qatar and Bahrain [6,9,11,17,20]. This affirms the institutional practice’s alignment with global standards and supports the validity of the established DRLCIs. However, integrating clinical outcome measures—such as diagnostic accuracy or radiologist confidence—would further validate the efficacy of lower-dose protocols and ensure that optimisation efforts do not compromise diagnostic quality.

Table 4 presents a comparative overview of radiation dose indices—Computed Tomography Dose Index Volume (CTDIvol) and Dose–Length Product (DLP)—for adult head CT examinations across different clinical indications (stroke, trauma, seizure, infection, and headache) from various international studies, including the current study. The table serves to contextualise the findings of this study within the broader landscape of global practices and highlights the variability in dose optimisation strategies between countries. For stroke imaging, CTDIvol values ranged from 43 mGy in Nigeria [12] to 66 mGy in Bahrain [17], with the current study reporting a value of 54.07 mGy. Similarly, DLP values varied considerably, from 879 mGy·cm in Nigeria to 1386 mGy·cm in the European Survey [21], with the current study aligning in the mid-range at 1205.78 mGy·cm. This suggests that local protocols in our setting are consistent with international benchmarks, neither overly conservative nor excessively high. Trauma-related CT doses displayed a similar pattern. The lowest CTDIvol was again reported in Nigeria [12] (43 mGy), while the highest (66 mGy) was observed in Bahrain. The DLP in Qatar was notably higher (1820 mGy·cm) [11], possibly due to extended scan lengths or multi-phase protocols, whereas the current study reported a balanced dose (54.06 mGy CTDIvol and 1205.74 mGy·cm DLP), indicative of dose-efficient imaging while maintaining diagnostic integrity. For seizure and infection evaluations, the current study reported relatively higher CTDIvol values (54.13 mGy and 54.15 mGy, respectively) compared to Nigeria’s significantly lower values (28 mGy and 34 mGy). However, the corresponding DLP values in our study (1212.66 and 1219.98 mGy·cm) remained within acceptable international ranges. These differences may reflect variations in image quality requirements, scanner technology, or protocol standardisation. In the case of headache, the current study’s values (CTDIvol: 54.07 mGy; DLP: 1205.78 mGy·cm) closely matched those from Bahrain, reinforcing the consistency of practice in this clinical context. Notably, CTDIvol values in the current study for all indications were relatively uniform (~54 mGy), suggesting a well-standardised institutional protocol across clinical scenarios.

## 5. Limitations

Despite the strength of the dataset and methodology, this study has limitations. First, it was a single-centre study utilising only one scanner model (GE Optima CT660), which may limit the generalisability of the results to other institutions with different scanner technologies or vendor-specific dose modulation algorithms. Second, only non-contrast CT head scans were included, excluding contrast-enhanced and multiphase examinations that are routinely used for vascular and oncological indications. This restricts the clinical relevance of the findings; future work should include these types of scans to capture a broader spectrum of clinical scenarios, as they often involve higher radiation doses and more complex imaging requirements. Finally, this study did not assess diagnostic image quality or clinical outcomes, which are essential to evaluating whether the radiation dose was appropriately matched to diagnostic need.

## 6. Conclusions

This study successfully established clinical indication-specific Diagnostic Reference Levels (DRLCIs) for adult head CT examinations using a robust dataset and standardised protocol. The findings demonstrate that while radiation dose metrics such as CTDIvol and DLP are relatively consistent across most clinical indications, subtle variations exist—particularly for complex cases such as infection and seizure—which justify the need for indication-specific benchmarking. The current study’s dose levels are comparable with international standards and fall within acceptable ranges, reinforcing the effectiveness of local dose optimisation practices. Implementing DRLCIs enhances the accuracy of dose monitoring, facilitates peer benchmarking, and supports the transition from anatomy-based to clinically justified imaging protocols in alignment with global recommendations from the ICRP and the EUCLID project [21,22].

## 7. Recommendations

To enhance the clinical utility and generalisability of DRLCIs, future studies should involve multi-centre collaborations across different regions and scanner platforms. This would allow for the development of national or regional DRLCI benchmarks. Additionally, incorporating contrast-enhanced examinations and evaluating image quality alongside dose metrics would provide a more comprehensive understanding of optimisation practices. Integration of artificial intelligence-assisted dose modulation, real-time dose monitoring, and image quality assessment tools is also recommended to support precision imaging. Finally, regular audit and review of DRLCIs should be encouraged as part of quality assurance programs to ensure continual adherence to the ALARA (As Low As Reasonably Achievable) principle and improve patient safety in radiological practices.

## Figures and Tables

**Table 1:** CT head scan protocol and exposure parameters.

Parameter	Head
Scan Type	Helical
Detector Rows	32
Pitch	0.531
Rotation Time (s)	0.5
Kilo Volt (kV)	120
Automatic Exposure Control Type	Smart mA
Milliampere (mA) Range (Manual mA)	130–440 (210)
Slice Thickness (mm) /Interval (mm)	5/5
Scanning Length (mm)	140–180

S: second; kV: kilo volt; mA: milliampere; mm: millimeter.

**Table 2:** Distribution of gender, BMI classification, and clinical indications among patients undergoing CT head examinations (*n* = 378).

Category	Variable	*n* (%)
Gender	Female	168 (44.4%)
	Male	210 (55.6%)
BMI Range	Healthy Weight (BMI * 18.5–24.9)	155 (41.0)
	Overweight (BMI 25–29.9)	179 (47.4)
	Obesity (BMI ≥ 30)	44 (11.6)
Indications	Trauma	102 (27.0)
	Headache	99 (26.2)
	Stroke	99 (26.2)
	Seizure	43 (11.4)
	Infection	35 (9.3)

* BMI: body mass index.

**Table 3:** Summary of radiation dose indices (CTDIvol and DLP (mGy.cm)) for CT head examinations by clinical indication, including mean, standard deviation, and 25th, 50th, and 75th percentiles.

Clinical Indication	Dose	Mean ± SD	75 Percentile
Headache	CTDI vol	51.26 ± 5.36	54.07
	DLP	1078.32 ± 176.24	1205.78
Infection	CTDI vol	52.90 ± 5.57	54.15
	DLP	1142.97 ± 127.71	1219.98
Seizure	CTDI vol	51.88 ± 6.10	54.13
	DLP	1099.97 ± 185.07	1212.66
Stroke	CTDI vol	50.65 ± 5.28	54.07
	DLP	1065.02 ± 194.73	1205.78
Trauma	CTDI vol	50.58 ± 5.59	54.06
	DLP	1052.52 ± 194.09	1205.74

CTDI vol: Computed Tomography Dose Index Volume; DLP: Dose–Length Product.

**Table 4:** Comparison of CTDIvol and DLP values for head CT examinations by clinical indication across studies [11,12,17,18,21].

Clinical Indication	Study	CTDIvol (mGy)	DLP (mGy·cm)
Stroke	European Survey	48	1386
	Nigeria	43	879
	Bahrain	66	1152
	Current Study	54.07	1205.78
Trauma	Nigeria	43	907
	Qatar	51	1820
	Bahrain	66	1286
	Current Study	54.06	1205.74
Seizure	Nigeria	28	995
	Current Study	54.13	1212.66
Infection	Nigeria	34	969
	Current Study	54.15	1219.98
Headache	Bahrain	67	1206
	Current Study	54.07	1205.78

## Data Availability

The data that support the findings of this study are available from the corresponding author upon reasonable request. Restrictions apply to the availability of these data due to privacy and ethical considerations.

## Abbreviations

The following abbreviations are used in this manuscript:

AEC	Automatic Exposure Control
ALARA	As Low As Reasonably Achievable
BMI	Body Mass Index
CTA	CT Angiography
CT	Computed Tomography
CTDIvol	Computed Tomography Dose Index Volume
DLP	Dose–Length Product
DRL	Diagnostic Reference Level
DRLCI	Diagnostic Reference Level based on Clinical Indication
EUCLID	European Study on Clinical Diagnostic Reference Levels
FOV	Field of View
GE	General Electric (as in GE Healthcare)
ICRP	International Commission on Radiological Protection
NDRL	National Diagnostic Reference Level
PACS	Picture Archiving and Communication System
RIS	Radiology Information System
SD	Standard Deviation
